# A novel scorpine-like peptide from the amazonian scorpion *Brotheas amazonicus* with cytolytic activity

**DOI:** 10.3389/fphar.2025.1652614

**Published:** 2025-09-03

**Authors:** Mouzarllem Barros Reis, Karla De Castro Figueiredo Bordon, Jonas Gama Martins, Gisele Adriano Wiezel, Ualter Guilherme Cipriano, Rudi Emerson de Lima Procópio, Vania Luiza Deperon Bonato, Eliane Candiani Arantes

**Affiliations:** ^1^ Department of BioMolecular Sciences, School of Pharmaceutical Sciences of Ribeirão Preto, University of São Paulo (USP), RibeirãoPreto, São Paulo, Brazil; ^2^ Graduate Program in Genetics, Conservation and Evolutionary Biology (PPG GCBEv), National Institute for Amazon Research (INPA), Manaus, Amazonas, Brazil; ^3^ Graduate Program in Basic and Applied Immunology, Ribeirão Preto Medical School, University of São Paulo (USP), Ribeirão Preto, São Paulo, Brazil; ^4^ Graduate Program in Biotechnology and Natural Resources of Amazon, University of the State of Amazonas (UEA), Manaus, Amazonas, Brazil

**Keywords:** *Brotheas amazonicus*, scorpine-like, breast cancer, antitumor, cytotoxic peptide, necrosis

## Abstract

**Introduction:**

Scorpion venoms contain bioactive molecules with potential antitumor properties. This study aimed to evaluate the cytotoxic effects of crude *Brotheas amazonicus* venom (BamazV) and its molecular weight–separated fractions on human breast cancer cell lines, with a focus on identifying active compounds and elucidating their mechanisms of action.

**Methods:**

Human breast epithelial (MCF10A) and breast cancer cell lines (SKBR3, MCF7, and MDA-MB-231) were first assessed for dose-dependent responses to paclitaxel, a standard chemotherapeutic agent. BamazV was fractionated by ultrafiltration into >10 kDa, 3–10 kDa, and <3 kDa fractions, which were tested for cytotoxic activity. The active fraction underwent reversed-phase chromatography, and the major bioactive peptide was characterized by mass spectrometry and Edman degradation. Cytotoxic mechanisms were investigated using cell death assays.

**Results:**

All cell lines showed a dose-dependent response to paclitaxel. Crude BamazV induced significant cytotoxicity at concentrations ≥ 50 μg/mL, with triple-negative MDA-MB-231 cells being the most sensitive. The >10 kDa fraction retained cytotoxic activity, leading to the isolation of a major peptide, BamazScplp1. Sequence analysis revealed 46–55% identity and 74–81% similarity to known scorpine-like peptides. Functional assays indicated that BamazScplp1 induced predominantly necrotic cell death, consistent with the activity profile of previously reported cytolytic scorpine-like molecules.

**Discussion:**

These findings identify BamazScplp1 as a scorpine-like peptide with selective cytotoxicity toward triple-negative breast cancer cells, underscoring the potential of *B. amazonicus* venom as a source of bioactive compounds for cancer research.

## 1 Introduction

The Amazon region is a biome encompassing several countries, with its largest territory located in Brazil (which contains approximately 60% of its area) (Monteiro et al., 2019; Martins et al., 2021). The Brazilian Amazon is home to a rich fauna and flora, with a notable diversity of scorpions (approximately 70 species described to date), with several species being of medical importance. Beyond the clinical implications of scorpion envenomation, this remarkable diversity also makes Amazonian scorpion venoms valuable sources of bioactive molecules with significant biotechnological potential (Martins et al., 2021; Martins et al., 2024; Reis et al., 2023).

Among the scorpion species of this biome, the scorpion *Brotheas amazonicus* (Lourenço, 1988) is an endemic species of the Amazon, having been reported only in the Brazilian state of Amazonas (Martins et al., 2021; Reis et al., 2023). It is a species with a coloration that varies from dark brown to black, with a reddish telson, ranging from 60–70 mm when adult, and can be found in urban and rural environments. Its venom has low toxicity, and accidents in humans are rare (when they occur, they have little clinical repercussion). In this scenario, the scorpion *B. amazonicus* is a species whose venom possesses significant biotechnological potential for the investigation of antimicrobial and antitumor actions, among others, and is currently underexplored in the scientific literature (Reis et al., 2023).

Scorpion venoms are complex biological mixtures composed of a wide array bioactive molecules, including peptides, proteins, enzymes, nucleotides, and low-molecular-weight organic compounds. While most of the scorpion peptides currently described exert their primary effects on ion channels, particularly sodium, potassium, and calcium channels, leading to autonomic manifestations commonly observed in envenomation (Isbister and Bawaskar, 2014; Pucca et al., 2015), evidence points to a broader pharmacological repertoire of venom-derived components. Beyond neurotoxicity, many of these peptides demonstrate antimicrobial, antiparasitic, immunomodulatory, and cytotoxic activities, highlighting their potential as versatile scaffolds for the development of novel therapeutic agents (Ahmadi et al., 2002; El-Qassas et al., 2024; Kwon et al., 2025; Reis and Arantes, 2024).

One notable example of the therapeutic potential of scorpion venom components is “scorpine”, a multifunctional peptide first described in the early 2000s following its isolation from the venom of the African scorpion *Pandinus imperator,* an African scorpion of the Chatidae family known for its low toxicity to humans. This 8.35 kDa peptide was initially characterized by its potent antibacterial and antimalarial activities (Conde et al., 2000). More recently, it has also been shown to block potassium ion channels and exert cytolytic effects on mammalian cells, including HEK 293 T cells (López-Giraldo et al., 2023). Since its discovery, several scorpine-like peptides have been identified in other scorpion species, reinforcing the pharmacological versatility and therapeutic relevance of this peptide family.

Given this expanding landscape of bioactivity, scorpion venom peptides have emerged as valuable candidates in the search for novel anticancer agents. Breast cancer remains one of the most prevalent and lethal malignancies worldwide, accounting for over 30% of all cases of neoplasia among women and exhibiting a global mortality rate of approximately 15% (Harbeck et al., 2019). Despite significant advances in early detection and molecular characterization, current therapeutic approaches, including surgery, radiotherapy, chemotherapy, and, more recently, immunotherapy (De Almeida et al., 2005), often lack specificity and are associated with high systemic toxicity. Most chemotherapeutic agents act by impairing fundamental cellular processes such as DNA replication and protein synthesis, which, while effective against rapidly dividing tumor cells, also severely affect normal proliferative tissues. These limitations underscore the urgent need for alternative therapeutic strategies that are both effective and selective (Harbeck et al., 2019).

In this context, venom-derived peptides, particularly those from scorpions, offer a promising avenue for the development of innovative cytotoxic agents with unique mechanisms of action. Expanding the understanding of their biological effects beyond ion channel interactions may open new perspectives for their application in oncology and other disease models.

In the present study, we demonstrated that the venom of the scorpion *B. amazonicus* contains a scorpine-like molecule with high cytolytic activity in human breast cancer tumor lines. The discovery of this molecule opens the field for research that makes it more selective for tumor cell lines and less specific for healthy cell lines, as well as a new possibility of use for scorpine-like peptides derived from scorpion venoms as a cancer therapy approach.

## 2 Materials and methods

### 2.1 Scorpion venom

The venom of the scorpion *Brotheas amazonicus* (BamazV, following the systematic nomenclature proposed by Delgado-Prudencio and collaborators) (Delgado-Prudencio et al., 2025) was obtained by extraction of 17 specimens (7 males and 10 females) collected in Manaus, capital of Amazonas, Brazil (3°03′03.0″S 59°59′52.9″W). Briefly, the animals were subjected to 10 V electrical stimulation in the telson region, with subsequent lyophilization of the venom pool and storage at −80 C until the experiment. The experiments were authorized by the Ministry of the Environment under SISBIO (Authorization for Research in Federal Conservation Units) permit no. 56748–1 and SISGEN (National System for Management of Genetic Heritage and Associated Traditional Knowledge) registration no. A4A9FDD.

### 2.2 Tumor cell line culture and cell treatment

The breast tumor cell lines SKBr3, MCF-7, and MDA-MB-231, as well as the control breast epithelial cell line MCF10A, were obtained from the American Type Culture Collection (ATCC, Manassas, VA) and maintained in RPMI 1640 (Roswell Park Memorial Institute) supplemented with 10% FBS and 1% penicillin/streptomycin at 37 C, 5% CO_2_. Cell death assay conditions were standardized using Paclitaxel on 4 cell lines. To standardize the cell death assay, 1 × 10^5^ cells of the 4 cell lines were seeded and treated 24 h later with Paclitaxel (0.01–1,000 µM). After 24 h of incubation, cell viability was measured using the MTT (3-(4,5-dimethylthiazol-2-yl)-2,5-diphenyltetrazolium bromide) assay (Supplementary Figure S1). Different concentrations of venom (2–250 μg/mL), its >10 kDa, 3–10 kDa, and <3 kDa fractions (0.2–25 μg/mL), respectively, Bamaz>10, Bamaz3-10, and Bamaz<3. Obtained through venom ultrafiltration (Supplementary Figure S2), and selected peaks from the >10 kDa fraction eluted on the RP-C18 column (0.0004–40 μg/mL, including major peaks and those with enough protein for bioassays, regardless of their abundance) were used to establish the IC_50_ curve (concentration required to achieve 50% of the maximum inhibitory effect) in cultures of healthy and tumor human cell lines. The samples were diluted in RPMI supplemented with 10% FBS and 1% penicillin/streptomycin and placed in culture for 24 h until analysis. RPMI was used as a negative control, and 30% DMSO was used as a positive control. Cells were seeded at a density of 1 × 10^5^ cells/well in a 96-well (Corning, 3,599) plate for stimulation. For all experiments, cells were left for 24 h for adhesion plus 24 h with stimuli before MTT or Flow Cytometry assays.

### 2.3 Fractionation of components present in the fraction >10 kDa from BamazV (Bamaz>10)

Bamaz>10 (7 mg, Supplementary Figure S2) was applied on a reversed-phase C18 column (10.0 mm × 250.0 mm, 5 μm, 300 Å, Jupiter^®^, Phenomenex, United States) using a Fast protein liquid chromatography (FPLC) system. The components adsorbed onto the resin matrix were eluted using a step concentration gradient from 0% to 100% of solution B (80% acetonitrile (MeCN) in 0.1% trifluoroacetic acid (TFA)) at a constant flow rate of 5 mL/min over 240 min. The gradient profile included an initial plateau of 0% for 4 min, followed by linear increases from 0% to 25% over 36 min, 25%–60% over 140 min, 60%–100% for 40 min, a final plateau of 100% maintained for 12 min and re-equilibration at 0% for 8 min. Absorbance was automatically registered at 214 nm by the FPLC Äkta Purifier UPC-10 system (GE Healthcare, Sweden), and fractions of 2.5 mL/tube were collected at 25 °C. Peak 59 (50 µg) was rechromatographed on a 2.1 mm × 250.0 mm C18 column (5 μm, 300 Å, Jupiter^®^, Phenomenex), eluting with a step concentration gradient from 0% to 100% of solution B, at a flow rate of 0.5 mL/min over 44 min. The gradient profile included an initial plateau of 0% for 8 min, followed by linear increases from 0% to 42% over 4 min, 42%–48% over 16 min, 48%–100% for 2 min, a final plateau of 100% maintained for 2 min and re-equilibration at 0% for 12 min. Absorbances (λ = 214 nm) were registered, and fractions of 0.5 mL/tube were collected at 25 °C.

### 2.4 Tricine-SDS-PAGE

*Brotheas amazonicus* crude venom (BamazV), its fractions (Bamaz>10, Bamaz3-10, Bamaz<3) (15 µg/well), and selected peaks (P9, P27, P28, P31, P32, P36, P57, P59, P75, P81) were dispersed in 20 µL of water and centrifuged at 13,000 *xg*, 4 °C, for 5 min. Protein concentration in the supernatant was estimated using a NanoDrop 2000 spectrophotometer (Thermo Scientific, United States) at 280 nm. Despite the limitations of UV absorbance for complex mixtures, this method was selected due to its low sample consumption and suitability for small-volume, low-yield venom fractions. A uniform extinction coefficient (ε_280_ = 1.0) was applied for relative quantification, and all measurements were performed in technical triplicate to ensure consistency. Soluble samples (3–15 µg of total protein/well) were prepared in Laemmli sample buffer containing final concentrations of 2% SDS (w/V), 10% glycerol, 5% β-mercaptoethanol, 0.002% bromphenol blue and 0.125 M Tris HCl, pH 6.8, Samples were heat-denatured at 95 °C for 5 min to ensure complete denaturation and reduction of disulfide bonds. Proteins were then separated onto a 10% T, 6% C separating Tricine Sodium Dodecyl Sulfate Polyacrylamide Gel Electrophoresis (Tricine-SDS-PAGE), overlaid by a 5% stacking gel. The stacking gel was prepared by mixing 200 µL of AB-6 stock solution (49.5% T, 6% C), 667 µL of 3× Tricine gel buffer (3 M Tris-HCl, 0.3% SDS, pH 8.45), and ultrapure water to a final volume of 2 mL (Schägger, 2006). Differences in protein loading reflect variation in protein yield among fractions and peaks; although equal loading was prioritized for comparative analyses, some samples (e.g., P75) were limited by available material. Electrophoretic separation was initiated and maintained at 60 V for 20 min, followed by a subsequent adjustment to 10 W/gel until the end of the electrophoretic run (Jiang et al., 2016). For molecular mass estimation, two commercial protein standards were used: Low Range Molecular Weight Marker (Sigma M3546; 1.06–26.6 kDa) and Precision Plus Protein™ Dual Color Standards (Bio-Rad #1610374; 10–250 kDa), allowing accurate visualization across a broad mass range. Gels were stained with 0.025% *Coomassie Blue* G-250, and the image was captured using a Gel Doc™ EZ Imager and the software Image Lab version 5.2.1 (Bio-Rad, United States).

### 2.5 Determination of cell viability by the MTT method

The viability of cells (MCF10A, SKBr3, MCF7, and MDA-MB-231) exposed to the fractions and/or peptides (crude venom, fractions <3, 3–10, and >10 kDa and peaks P9, P27, P28, P31, P32, P36, P57, P59, P75, P77, P81, AND P85) was determined by the MTT reduction method. After 24 h of treatment, the wells received 10 μL of MTT (5 mg/mL) and were incubated for 4 h at 37 °C. After incubation, plates were centrifuged at 400 *g* for 5 min, the supernatant was discarded by inversion. Formazan crystals were solubilized in 100 μL of DMSO (dimethyl sulfoxide) under orbital shaking for approximately 20 min. The absorbance of the wells at 570 nm was then read. The absorbance results of the control groups (treatment with culture medium only) corresponded to 100% cell viability, and the groups treated with peptides had their viability percentages calculated from the control. All the experiments were performed in triplicate and were independently repeated at least 3 times.

### 2.6 Cell death assay and labeling for cytometry

Cells were seeded at 1 × 10^5^ concentration in 96-well (Corning, 3,599) plates and left overnight for adhesion. The cells were treated with BamazScplp1 (50 μg/mL) for 24 h. The cells were then released from the plate and transferred to round bottom 96-well plate to start labeling with Annexin V (eBioscience, 88–8,103–74) and Fixable Viability Stain 780 (FVS) (eBioscience, 65–0865–14). For labeling, the cells were washed with Phosphate buffered saline (PBS) and labeled with FVS 1:1000 for 15 min at room temperature, after which the cells were washed with Annexin V Binding Buffer (Invitrogen) and labeled with Annexin V diluted in Annexin V Binding Buffer for 15 min at 4 °C protected from light. The samples were passed through the BD FACSCanto™ II equipment. Analyses were performed using FlowJo software version 10 (Becton Dickinson and Company, Franklin Lakes, NJ, United States). 10,000 events were acquired for all cells. Cells were gated by size (FSC) and granularity (SSC), singlets (FSC-A X FSC-H), followed by Annexin + FVS- (apoptosis) and Annexin-FVS+ (necrosis) (Supplementary Figure S6).

### 2.7 Characterization of BamazScplp1 eluted in P59 from Bamaz>10

The initial 43 amino acid residues from the N-terminal region of the rechromatographed peak 59 were determined through Edman degradation (Edman and Begg, 1967) using an automated PPSQ-33A sequencer (Shimadzu Co., Japan). Sequence analysis was performed with BLAST (Basic Local Alignment Search Tool). Scorpine-like peptide sequences were retrieved from the Universal Protein Resource Knowledgebase (www.uniprot.org). Sequence alignment was created by the MultAlin Interface Page (Corpet, 1988), and the figure was generated by the ESPript server (Gouet et al., 1999).

Rechromatographed peak 59, hereafter designated BamazScplp1 (Bamaz-scorpine-like-1), was named following the systematic nomenclature proposed by Delgado-Prudencio et al. (2025) and the naming convention reported at UniProt entry P0C8W5 (Hg-scorpine-like-2). The sample was resuspended in TA50 (50% (V/V) MeCN in 0.1% (V/V) TFA), mixed at 1:1 (V/V) ratio with a saturated matrix solution of sinapinic acid (SA) in TA50, and subjected to matrix-assisted laser desorption/ionization (MALDI) time-of-flight (TOF) mass spectrometry analysis using an UltrafleXtreme system (Bruker Daltonics, United States). Mass spectra were acquired in positive reflector mode with either 500 or 2,500 laser shots per spectrum (single position), following external calibration with Bruker Protein Calibration Standard I (mass range: 4,000–20,000 Da; Bruker Daltonics). Mass spectrometry analyses (MS and MS/MS) were performed in two independent technical replicates derived from the same sample (P59), each spotted at randomized positions on the MALDI target plate. These replicates were used to confirm the analytical reproducibility of mass measurements and peptide fragmentation patterns. Data were processed using flexAnalysis software, version 3.4.76.0(Bruker Daltonics).

*Coomassie*-stained gel bands of P59 were excised from Tricine-SDS-PAGE gels and destained with 50% (V/V) methanol in 50 mM ammonium bicarbonate (pH 8). Samples were reduced with 10 mM dithiothreitol (DTT) at 56 °C for 40 min under agitation (600 rpm), followed by alkylation with 55 mM iodoacetamide at 25 °C in the dark for 1 h. The gel pieces were then dehydrated with acetonitrile and dried.

In-gel digestion was performed by incubating each sample with 40 ng of sequencing-grade modified trypsin (Promega V5111) in 4 µL of 50 mM ammonium bicarbonate, at 37 C for 15 h under continuous agitation (600 rpm). The digestion was stopped by adding 1% (V/V) TFA. Resulting peptides were desalted using ZipTip^®^ Pipette Tips with 0.6 µL C18 resin (MilliporeSigma, United States), and eluted in 50% acetonitrile containing 0.1% TFA.

For MALDI-TOF mass spectrometry analysis, desalted peptide samples were mixed at a 1:1 (V/V) ratio with a saturated solution of α-cyano-4-hydroxycinnamic acid (HCCA; 10 mg/mL in TA50) and spotted onto a polished steel target plate. Spectra were acquired in positive reflector mode with 1,500 laser shots per spectrum, following external calibration using the Bruker Peptide Calibration Standard (mono-isotopic, 1,000–3,500 Da; Bruker Daltonics). The method yielded a mass resolution exceeding 14,000 and a mass accuracy below 50 ppm. Data were acquired from multiple technical replicates at randomized target positions to ensure analytical reproducibility and robustness.

Fragmentation of the ten most intense precursor ions was performed by MALDI-TOF/TOF using post-source decay in LIFT mode without collision gas. Each fragmentation spectrum was generated from 2,500 laser shots.

Raw MS and MS/MS data were processed using MASCOT MS/MS Ions Search (https://www.matrixscience.com/cgi/search_form.pl?FORMVER=2& SEARCH=MIS) with a precursor mass tolerance of 1.2 Da and a fragment mass tolerance of 0.6 Da. Carbamidomethylation of cysteine was set as a fixed modification; and oxidation (Met/His/Trp), Gln- > pyro-Glu (N-term Q/N-term E), and deamidated (Asn/Gln) were considered variable modifications. Results were searched against the SwissProt database (taxonomy: ‘Metazoa’). Additionally, all spectra were manually investigated and manual *de novo* sequencing was validated using the MS-Product tool (https://prospector.ucsf.edu/prospector/cgi-bin/msform.cgi?form=msproduct).

### 2.8 Statistical analysis

All data represent mean ± standard deviation. Data were analyzed using one-way ANOVA with Bonferroni posttest for multiple groups or Student’s t-test for two-group comparisons. Data were considered statistically significant when *p* < 0.05. Information about replicates and independent experiments for each analysis are included in figure legends.

## 3 Results

### 3.1 Response of tumor cells to crude venom and chemotherapy

**FIGURE 1:**
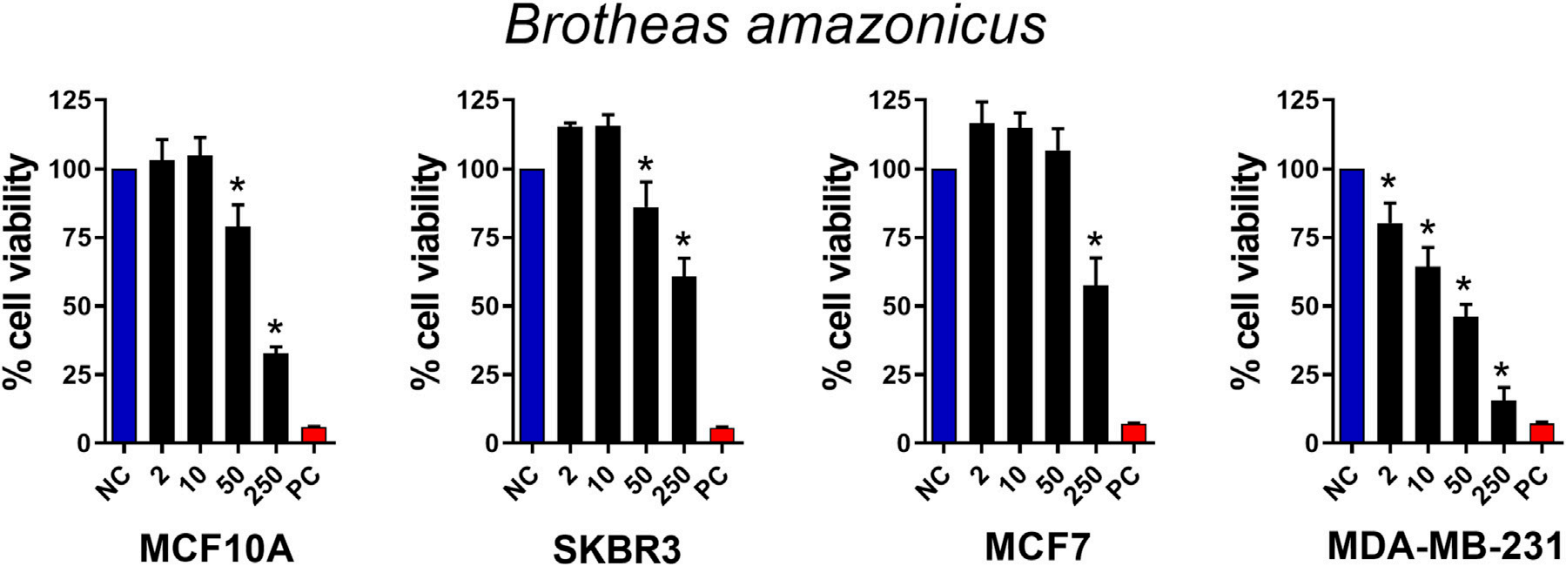
Antitumor activity of *Brotheas amazonicus* scorpion crude venom. MCF10A, SKBR3, MCF7, and MDA-MB-231 cell lines (seeded at 1 × 10^5^ cells/well) were treated and incubated with venom concentrations of 2, 10, 50, and 250 μg/mL for 24 h. Then, cell viability was assessed using the MTT assay. Data represent the mean ± SD of triplicate measurements from 4 independent experiments. NC - negative control (only culture medium). PC - positive control (30% DMSO). Statistical significance: group vs. NC, *p < 0.05.

As a means of validating the chemosensitivity of the selected breast cancer cell lines, we first tested their response to paclitaxel at concentrations of 0.01–1,000 μM, a chemotherapeutic drug widely used in the clinical treatment of breast cancer. As shown in Supplementary Figure S1, all cell lines used responded in a dose-dependent manner to paclitaxel. All cell types responded to paclitaxel stimulation. MCF10A and SKBr3 responded at doses of 1 μM and above, while MCF7 and MDa-MB-231 responded at doses of 10 μM and above. In the next experiment, we used crude venom from the *B. amazonicus* scorpion at concentrations of 2, 10, 50 and 250 μg/mL (Figure 1). Most cells responded with death at 50 μg/mL and above, with the triple negative cell line (MDA-MB-231) being the most susceptible, responding at all venom doses. The Selectivity Index (SI) (IC_50_ no cancer cell/IC_50_ cancer cell) was 0.416, 0.5, and 2.5 for SKBr3, MCF7, and MDA-MB-231 respectively. This experiment demonstrated that crude venom from the *B. amazonicus* scorpion showed cytotoxic activity in tumor cells, comparable to paclitaxel, suggesting potential antitumor effects that warrant further investigation.

### 3.2 Response of tumor cells to weight-separated fractions

**FIGURE 2:**
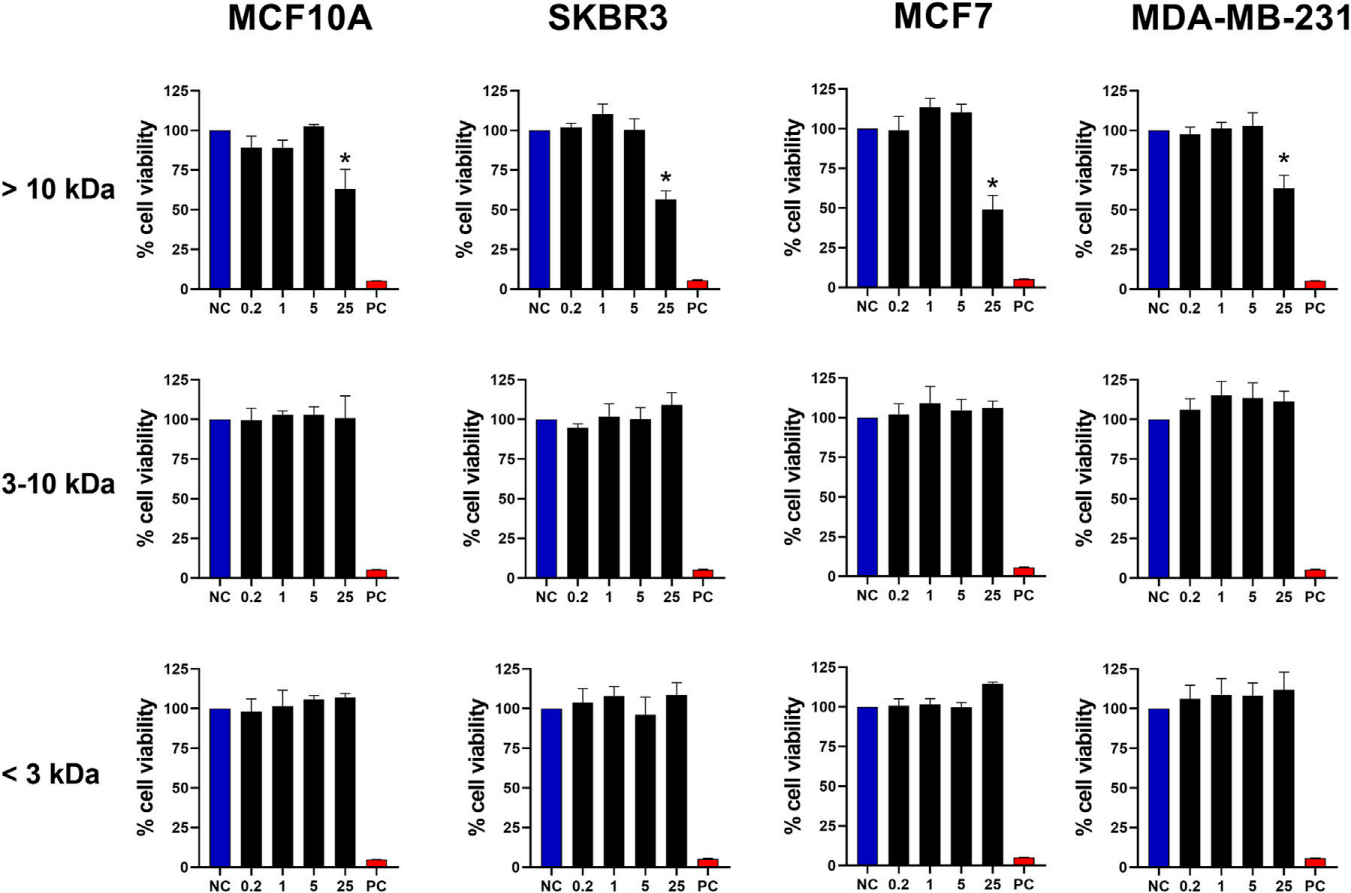
Antitumor activity of fractions from BamazV separated by molecular weight. Fractions were separated through an ultrafiltration process using a regenerated cellulose membrane (Amicon^®^ Ultra-15 Centrifugal Filter Ultracel^®^ 10K and 3K). MCF10A, SKBR3, MCF7, and MDA-MB-231 cell lines (seeded at 1 × 10^5^ cells/well) were treated and incubated with BamazV fractions (<3 kDa, 3–10 kDa, and >10 kDa) at concentrations of 0.2, 1, 5, and 25 μg/mL for 24 h. Then, cell viability was assessed using the MTT assay. Data represent the mean ± SD of triplicate measurements from 3 independent experiments. NC - negative control (only culture medium). PC - positive control (30% DMSO). Statistical significance: group vs. NC, *p < 0.05.

To refine our investigation and determine the molecular weight range of the molecule responsible for the cytotoxic activity observed in BamazV, we subjected the dispersed venom to an ultrafiltration process, which fractionated the sample into distinct molecular weight ranges, as shown in Supplementary Figure S2. Thus, we stimulated breast tumor cells with fractions >10 kDa, 3–10 kDa and <3 kDa derived from the crude venom. As observed in Figure 2, only the fraction >10 kDa maintained its cytotoxic activity, promoting the death of almost 50% of the cells with a dose of 25 μg/mL. These results demonstrate that the molecule with cytotoxic activity present in BamazV probably has a weight greater than 10 kDa. This finding indicates that the active component is likely a protein, which supports the application of protein-specific strategies for its purification and structural characterization. Initial isolation may involve chromatographic techniques or electroelution from SDS-PAGE if the activity can be associated with a specific band. Subsequent characterization could include mass spectrometry to determine molecular weight, sequence identification via LC-MS/MS or Edman degradation, and analysis of tertiary structure using circular dichroism, NMR spectroscopy, or other high-resolution methods.

### 3.3 Bamaz>10 kDa fractionation

**FIGURE 3:**
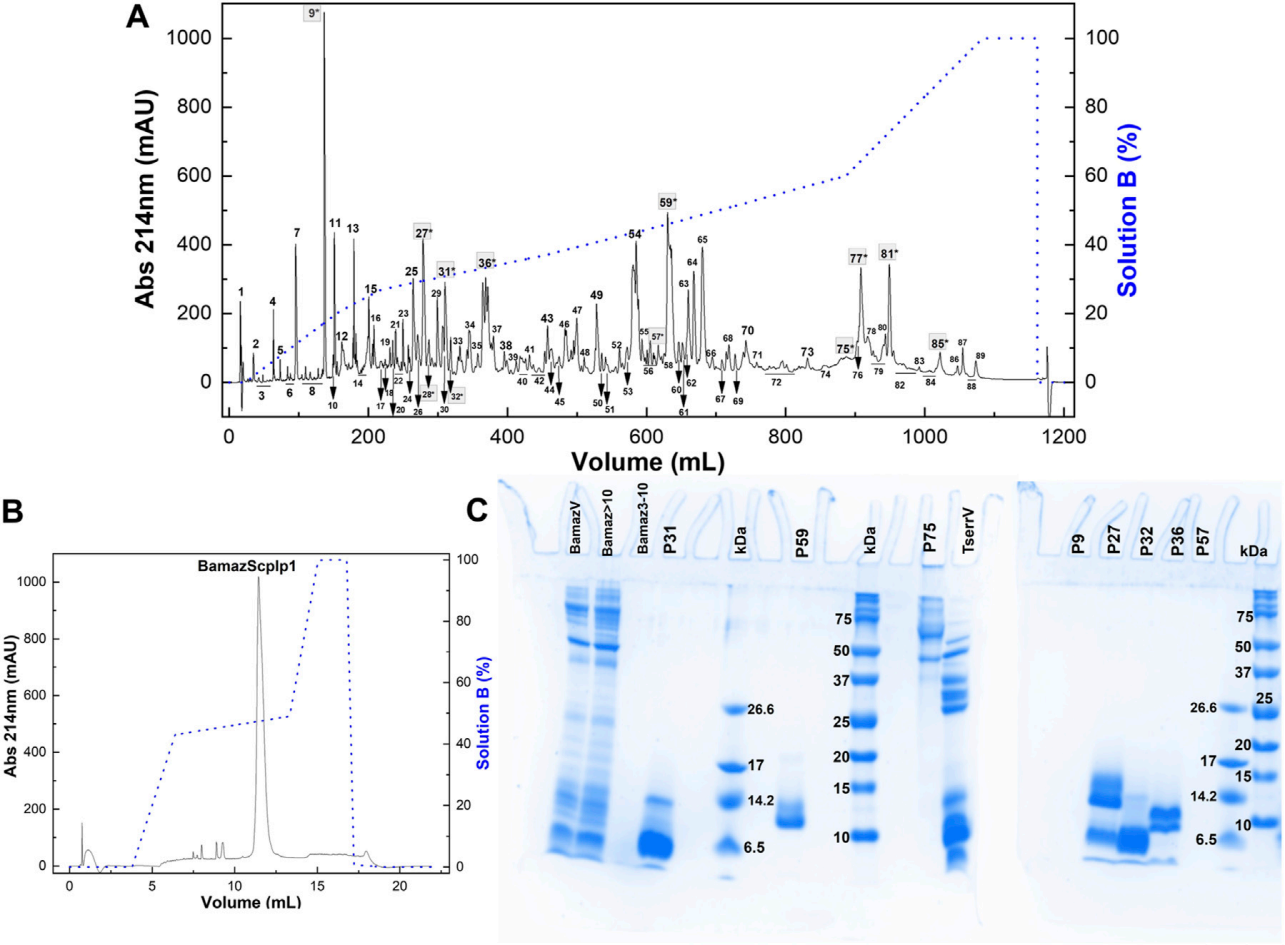
**(A,B)** Chromatographic profiles of fraction >10 kDa from *Brotheas amazonicus* venom (Bamaz>10) using an RP-FPLC system. Protein elution was performed using a segmented gradient from 0% to 100% of solution B (80% acetonitrile in 0.1% trifluoroacetic acid), represented by the blue line, with absorbance monitored at 214 nm and 25 °C. **(A)** Bam>10 (7 mg) was eluted on a C18 column (250 × 10 mm, 5 μm, 300 Å) at 5 mL/min, and peaks are numbered. **(B)** Peak 59 (50 μg) was rechromatographed on a C18 column (250 × 2.1 mm, 5 μm, 300 Å) at 0.5 mL/min. **(C)** Electrophoretical profile of *Brotheas amazonicus* venom (15 µg), Bamaz>10 (15 µg) and Bamaz3-10 (3 µg) fractions, and selected peaks compared to *Tityus serrulatus* venom (15 µg). P31 (8 µg), P59 (8 µg), P75 (2.7 µg), P9 (42 µg), P27 (10 µg), P32 (8 µg), P36 (12 µg), P57 (8 µg). kDa: molecular mass markers (5 μL, Sigma M3546–1,060–26,600 Da; Bio-Rad #1610374–10–250 kDa). Gels were stained with *Coomassie Blue* G-350.

Bamaz>10 was subjected to reversed-phase fast protein liquid chromatography (RP-FPLC) on a C18 column (250 mm × 10 mm). Its major peak, fraction P59 (Figure 3A), representing 14.3% of the total soluble protein in the >10 kDa fraction, was further purified by rechromatography on a different C18 column (250 × 2.1 mm). The resulting pure toxin, which constituted 79.7% of P59 (Figure 3B), was designated BamazScplp1 and accounted for 11.4% of the total protein in Bamaz>10. SDS-PAGE analysis revealed distinct protein banding patterns, indicating the presence of multiple proteins in the different eluted peaks (Figure 3C). The electrophoretic profile of Bamaz>10 closely resembled that of BamazV, suggesting a similar protein composition. The detection of proteins smaller than 10 kDa in the >10 kDa fraction indicates that the ultrafiltration process using a 10 kDa regenerated cellulose membrane (Amicon^®^ Ultra 15) did not achieve complete size-based separation. This limitation is consistent with the known variability around membrane cutoff thresholds and may be exacerbated by nonspecific adsorption or conformational flexibility of small proteins, which can facilitate their retention or passage through the membrane under centrifugal conditions. Nevertheless, the ultrafiltration step was intended as a preparative enrichment strategy, and the major cytotoxic component, BamazScplp1, was consistently recovered in the >10 kDa fraction, as confirmed by SDS-PAGE and mass spectrometry, supporting the reliability of the downstream analyses. Notably, no bands were detected in the Bamaz3-10 fraction, possibly due to a low sample amount or limited staining efficiency. P59 exhibited two protein bands within the 10–14.2 kDa range. Several peaks exhibited protein bands within the 6.5–14.2 kDa range, while P27 also contained bands above 14.2 kDa (Figure 3C). The lane of P75, loaded with 2.7 µg of protein, displayed at least four distinct protein bands above 37 kDa. In contrast, P57 showed no visible bands, despite loading approximately three times the amount used for P75, which was clearly detected in the gel. Similarly, no bands were observed for P9, even with a high protein load of 42 µg.

### 3.4 Investigation of chromatogram peaks with potential antitumor activity

**FIGURE 4:**
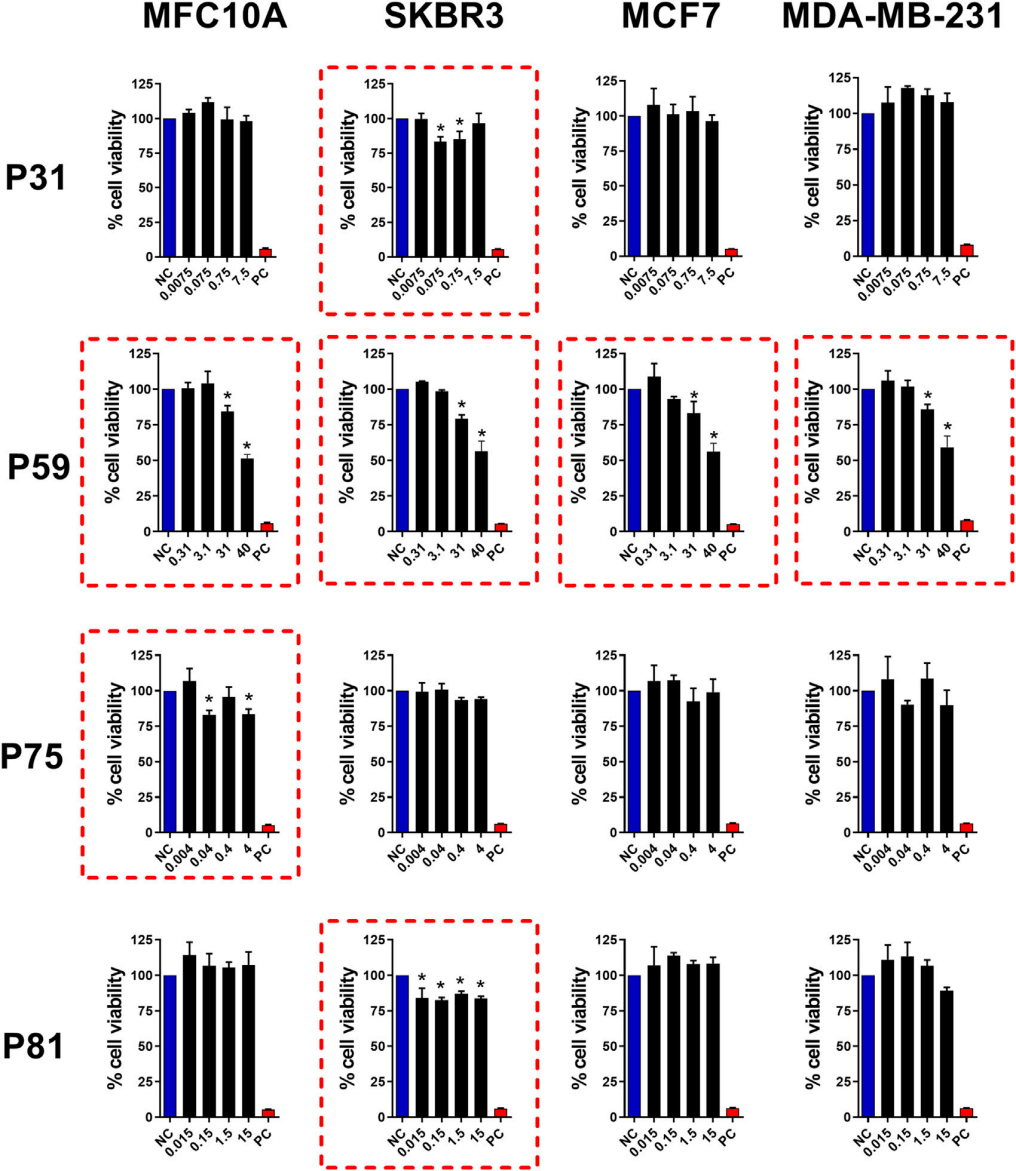
Antitumor activity of FPLC peaks from Bamaz>10 fraction. Peaks P31, P59, P75 and P81 were tested for antitumor effects in breast cancer cell lines. MCF10A, SKBR3, MCF7, and MDA-MB-231 cell lines (seeded at 1 × 10^5^ cells/well) were treated and incubated with the indicated doses (see graphs) for 24 h. Then, cell viability was assessed using the MTT assay. Data represent the mean ± SD of triplicate measurements from 4 independent experiments. NC - negative control (only culture medium). PC - positive control (30% DMSO). Statistical significance: group vs. NC, *p < 0.05.

Following chromatographic separation of the >10 kDa fraction, we investigated the cytotoxic potential of individual peaks against breast-derived cell lines. As shown in Figure 4, four peaks exhibited significant cytotoxic effects. Peaks 31 and 81 selectively reduced the viability of the SKBr3 cell line at concentrations of 0.075 μg/mL and 0.015 μg/mL, respectively. In contrast, peak 75 demonstrated selective cytotoxicity only toward the non-tumorigenic MCF10A cell line at 0.04 μg/mL. The low activity levels exhibited by peaks P31, P75 and P81 may be due to high toxicity and selectivity, deserving further studies to elucidate their mechanism of action. Notably, peak 59 induced cytotoxicity across all tested tumor cell lines (SKBR3, MCF7, and MDA-MB-231), as well as the control cell line MCF10A (at 31 μg/mL or higher). Additional peaks–P9, P27, P28, and P32 (Supplementary Figure S3); P36, P57, P77, and P85 (Supplementary Figure S4) – showed no detectable cytotoxic activity under the tested conditions. Based on its broader spectrum of activity, peak 59 was selected for further characterization to elucidate its molecular properties and mechanisms of action related to cell death induction.

### 3.5 Characterization of BamazScplp1

**FIGURE 5:**
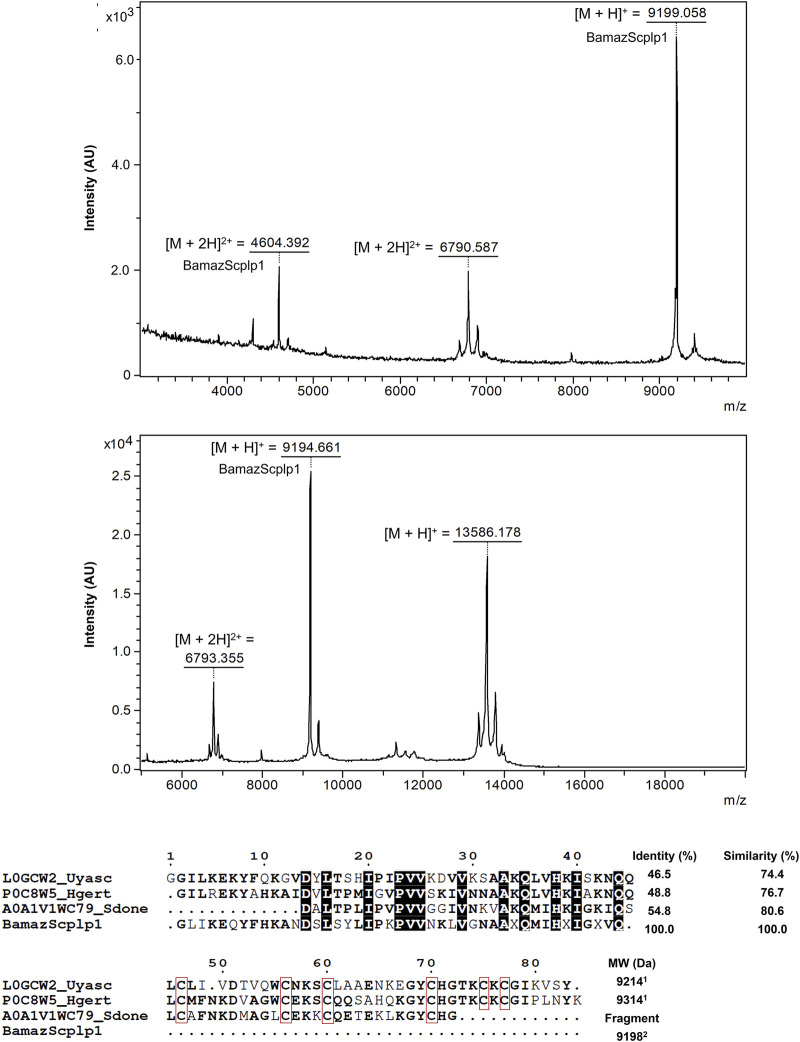
Structural and sequence analysis of P59 from Bamaz>10 fraction. (A,B) MALDI-TOF mass spectra of P59 acquired in positive reflector mode using sinapinic acid (SA) matrix in the ranges of 3–10 kDa (2,500 shots/spectrum) (A) and 5–20 kDa (500 shots/spectrum) (B). (C) Sequence alignment of the partial N-terminal sequence of BamazScplp1 (43 amino acids) with scorpine-like peptides. Scorpine-like-2 (*Urodacus yaschenkoi*, L0GCW2), Hg-scorpine-like-2 (*Hoffmannihadrurus gertschi*, P0C8W5), and a putative scorpine-like peptide (*Superstitionia donensis*, A0A1V1WC79). Conserved residues are bolded, highly conserved residues are highlighted with black squares, and cysteine residues are enclosed in red squares. Alignment was generated using the MultAlin server (Corpet, 1988), and the figure was rendered with ESPript (Gouet et al., 1999). Identity and similarity percentages were calculated using the LAlign tool (https://www.ebi.ac.uk/jdispatcher/psa/lalign). ^1^Molecular weight of the oxidized monoisotopic toxin (S–S) calculated by the Sequence Editor 3.2 program; ^2^Determined by mass spectrometry.

The mass spectrum of P59 showed a major peak intensity at an average ion m/z 9,199 Da ([M + H]^+^), along with an ion at m/z 4,604 Da ([M + 2H]^2+^; Figure 5A), designated BamazScplp1. Additionally, P59 included another peak at an average ion m/z 13,586 Da and an ion at m/z 6,793 Da ([M + 2H]^2+^; Figure 5B). The first 43 N-terminal amino acids from BamazScplp1 were determined by Edman degradation method as GLIKEQYFHKANDSLSYLIPKPVVNKLVGNA AHQMIHGIGGVQ. This N-terminal sequence corresponds to 50.9% of the molecule.

In addition, the internal peptide EQYFHK from BamazScplp1 (Supplementary Figure S5) was identified by *de novo* sequencing. Although additional peptide sequences could not be reliably deduced due to suboptimal fragmentation patterns, the complete mass fingerprint is provided in Table 1. The protein sequence data of BamazScplp1 reported in this paper will appear in the UniProt Knowledgebase under the accession number C0HME9. Sequence alignment revealed 46%–55% identity and 74%–81% similarity with scorpine-like sequences deposited in public databases, including: a putative scorpine-like peptide from *Superstitionia donensis* (A0A1V1WC79), Hg-scorpine-like-2 from *Hoffmannihadrurus gertschi* (P0C8W5), and scorpine-like-2 from *Urodacus yaschenkoi* (L0GCW2) (Figure 5C).

**TABLE 1:** Mass fingerprint obtained from in-gel tryptic digestion of P59 protein bands.

Apparent MW (kDa)	*m/z*
59.1	870.5417; 914.4554; 1,170.5267; 1,249.4853; 1,298.5059; 1,314.5005; 1,742.6054; 1,786.6978; 2,239.1013; 2,583.0173; 2,663.2330; 2,807.2791; 3,346.5955
59.2	712.2642; 842.5572; 851.4557; 1,170.6094; 1,208.5651; 1,623.9546; 1,645.9477; 1,661.9198; 1,712.8454; 1,769.8735; 2,239.1607; 2,807.3548

### 3.6 BamazScplp1 mechanism of death

**FIGURE 6:**
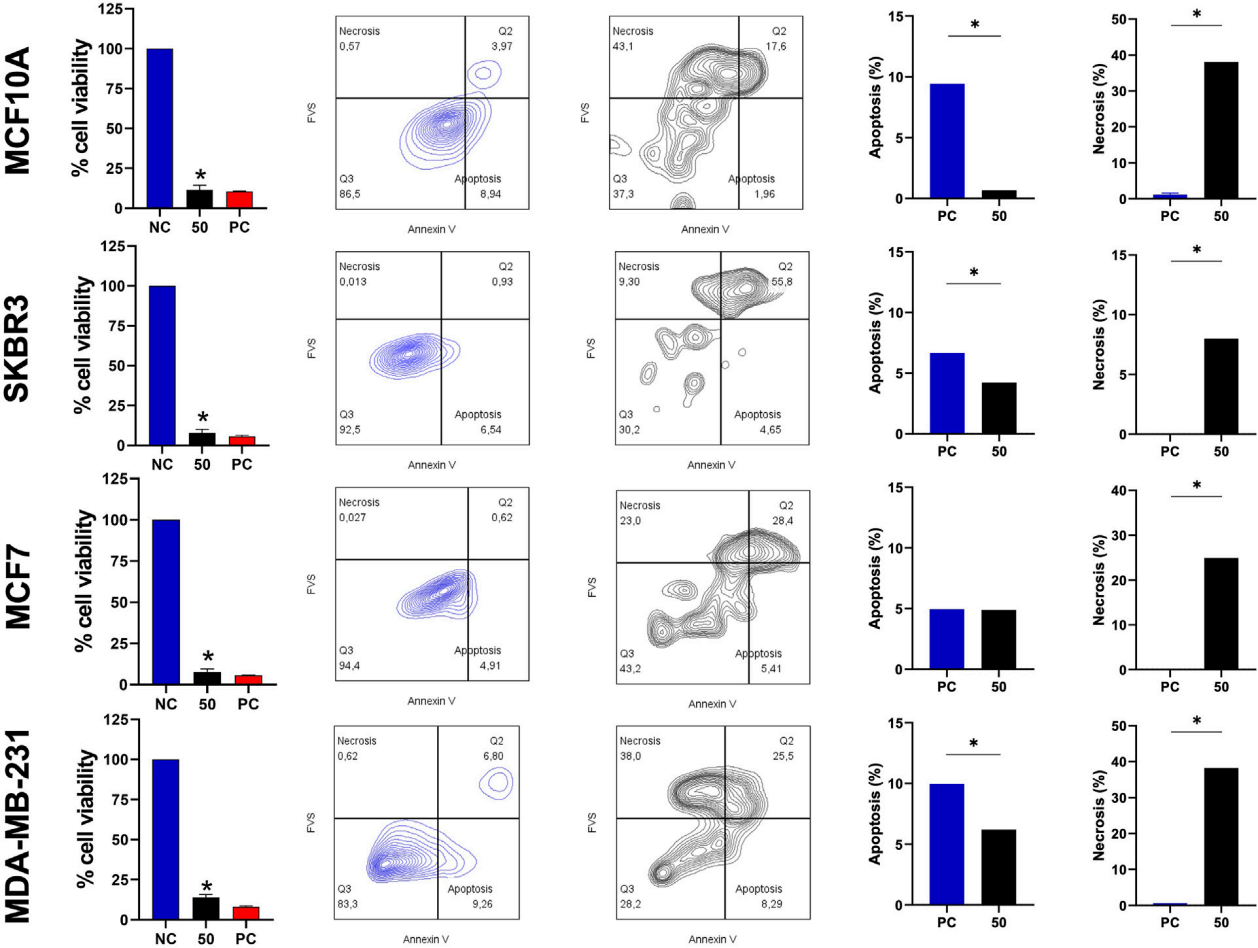
Viability assay and type of cell death after treatment with the scorpine-like peptide. MCF10A, SKBR3, MCF7, and MDA-MB-231 cell lines (seeded at 1 × 10^5^ cells/well) were treated and incubated with BamazScplp1 (50 μg/mL) or controls (NC: culture medium; PC: 30% DMSO) for 24 h. From left to right, cell viability was assessed using the MTT assay. Representative flow cytometry dot plots (NC vs. BamazScplp1-treated cells) and quantification of apoptotic and necrotic cell death after treatment. Data represent the mean ± SD of triplicate measurements from 1 experiment. NC - negative control (only culture medium). Statistical significance: group vs. NC, *p < 0.05.

Considering the cytolytic activity of BamazV (Figure 1), we addressed the question whether BamazScplp1 would induce cell death by apoptosis or necrosis. All cell lines were treated with 50 μg/mL BamazScplp1 for 24 h (Figure 6). After treatment, the cells were assessed for viability and type of cell death. MCF10A cells showed significantly reduced viability after peptide exposure, with decreased apoptosis and a marked increase in necrosis compared to the untreated control. The same phenotype can be observed for the SKBR3 and MDA-MB-231 strains. The MCF7 strain showed reduced cell viability and increased necrotic cell death following treatment. However, no significant difference in apoptosis was observed compared to the untreated control. These findings indicate that necrosis is the major mechanism of cell death induced by BamazScplp1, consistent with reports in the literature describing other scorpine-like molecules with high cytolytic activity.

## 4 Discussion

This study reports the identification and partial characterization of BamazScplp1, a novel scorpine-like peptide isolated from the venom of *Brotheas amazonicus* (BamazV), exhibiting potent cytolytic activity against human breast cancer cell lines. Most studies on scorpion venoms have centered on their clinical relevance, primarily addressing the epidemiological aspects and pathophysiological effects of envenomation by species of medical relevance, rather than on the discovery of bioactive molecules (Martins et al., 2021; Reis et al., 2023). Our findings highlight the potential of BamazV as a source of new therapeutic leads, especially in oncology, where novel cytotoxic agents are urgently needed.

Animal venoms are rich sources of new drug candidates, with a classic example being captopril, an antihypertensive drug derived from snake venom peptides (Bordon et al., 2020).

This study is significant for two main reasons: it identifies a previously unknown molecule from the scorpion *B. amazonicus* and demonstrates its cytolytic activity against human breast cancer cell lines. Supplementary Figure S1, 1 show that paclitaxel, a standard chemotherapeutic agent, and the crude venom of *B. amazonicus* have comparable effects, highlighting the venom’s potential for alternative anticancer therapies.

Notably, BamazScplp1 demonstrated strong cytolytic activity against the triple-negative MDA-MB-231 breast cancer cell line, a highly aggressive subtype, associated with rapid disease progression to death and limited treatment options due to the absence of estrogen, progesterone or HER2 receptors (the current targets of breast cancer therapies) (Akshata Desai, 2012; Zagami and Carey, 2022; Xiong et al., 2024). Of importance, the cytolytic activity of scorpine-like peptides is mainly attributed do the n-terminal sequence, which forms a helix when interacting with the membrane. The removal of n-terminal reduces cytolytic activity in other scorpine-like peptides (López-Giraldo et a., 2023).

However, like many conventional chemotherapeutics, BamazScplp1 lacks selectivity between tumorigenic and non-tumorigenic cells (MCF10A). To overcome this limitation, future strategies may include conjugation with tumor-targeting antibodies or encapsulation in selective delivery systems such as PLGA nanoparticles (Acharya and Sahoo, 2011; Rezvantalab et al., 2018; Saraf et al., 2021; Shen et al., 2025). These approaches have shown success in targeted therapy, as exemplified by antibody-drug conjugates to improve their specificity (Thomas et al., 2016; Yang et al., 2023; Shi et al., 2025). A successful example is Trastuzumab, an antibody targeting the HER2 protein expressed in some types of tumors. This antibody has been used as a carrier for cytolytic compounds, such as Trastuzumab-emtansine (T-DM1) and trastuzumab-deruxtecan (T-DXd), among others (Haddley, 2013; Hurvitz et al., 2013; Baron et al., 2014; Krop and Winer, 2014; von Arx et al., 2023; Zhang et al., 2024).

Electrophoretic and mass spectrometric analyses provided insights into the biochemical diversity of BamazV. Fractions P27, P31, and P32 (Figure 3C) displayed bands in the <6.5 kDa–10 kDa range, consistent with small ion channel toxins (NaTx, KTx, CaTx), antimicrobial peptides, and hypotensins, common components in scorpion (Alvarenga et al., 2012; De Oliveira et al., 2018; Monteiro et al., 2019). Protein bands in the 10–15 kDa range may include phospholipases A_2_, lysozymes, and protease inhibitors. MALDI-TOF analysis confirmed the presence of two major components in P59: a ∼9.2 kDa peptide identified as BamazScplp1 and a ∼13.5 kDa co-purified protein (Figures 5A,B). Densitometry (data not shown) of the two protein bands (Figure 3C) revealed that BamazScplp1 accounts for approximately 63% of the total band intensity, indicating it is the predominant component within peak 59.

Ongoing studies are focused on the comprehensive biochemical and structural characterization of the 13.5 kDa co-purified protein. Detailed findings will be presented in a forthcoming publication.

The BetaSPN-type CS-αβ motif is a conserved structural fold found in β-family scorpine-like peptides from scorpion venom, characterized by a compact cysteine-stabilized scaffold comprising a short α-helix and a triple-stranded antiparallel β-sheet, typically stabilized by disulfide bridges (C1–C4, C2–C5, C3–C6). As part of the broader CS-αβ superfamily, this motif confers high thermodynamic stability, resistance to proteolysis, and multifunctionality, including antimicrobial and cytolytic activities. Sequence analysis of BamazScplp1 showed high similarity (46%–55% identity, 74%–81% similarity) to scorpine-like peptides from *Superstitionia donensis*, *Hoffmannihadrurus gertschi*, and *Urodacus yaschenkoi* (Figure 5C). Pairwise alignments yielded significant Waterman-Eggert scores (up to 140) and highly significant E-values (<5.9 × 10^−12^), indicating that the observed homology is statistically robust and unlikely to have occurred by chance. These findings, together with the presence of the CS-αβ motif, suggest that BamazScplp1 may share the dual antimicrobial and cytolytic properties characteristic of scorpines, making it a promising lead for therapeutic development.

High-molecular-weight proteins observed in P75 (>37 kDa) align with enzymatic components previously reported in *Tityus* spp., such as amidating enzymes, amylase, chitinase, hyaluronidase, lipase, metalloproteases, phosphodiesterase, and angiotensin- or endothelin-converting enzymes (for review see Wiezel et al., 2024). The absence of aggregation artifacts and the chromatographic resolution reflect genuine abundance, underscoring the biochemical diversity of the venom and the importance of assessing distinct molecular weight ranges in bioactivity-guided fractionation.

The electrophoretic profile of Bamaz>10 closely resembled unfractionated BamazV, indicating minimal loss of low-molecular-weight components during ultrafiltration. In contrast, no visible bands were detected in Bamaz3-10 or P9 (Figure 3C), possibly due to low protein yield or limitations of *Coomassie* staining, which relies on electrostatic interactions with basic amino acids (arginine, lysine, and histidine) and can be hindered by post-translational modifications or low hydrophobic content (Steinberg, 2009). Similarly, P57 showed no detectable bands despite higher loading, reinforcing the idea that certain venom components may not stain efficiently.

In summary, our results provide the first molecular characterization of a bioactive component from *Brotheas amazonicus* venom, identifying BamazScplp1 as a promising candidate for future drug development. This work underscores the importance of exploring underrepresented venom sources and highlights the potential of scorpion-derived peptides in biomedical applications.

## 5 Conclusion and perspectives

This study reports, for the first time, the identification and partial molecular characterization of BamazScplp1, a scorpine-like peptide from *Brotheas amazonicus* venom. This discovery broadens our understanding of the molecular diversity in non-buthid scorpions and highlights their untapped potential as sources of bioactive molecules. The cytolytic effect of BamazScplp1 on human breast cancer cells positions it as a promising lead compound for anticancer drug development.

To enhance therapeutic potential and minimize off-target effects, future strategies may include its encapsulation in biodegradable polymeric carriers (e.g., PLGA nanoparticles) or its conjugation with monoclonal antibodies to generate antibody–drug conjugates (ADCs). Such approaches aim to improve specificity toward tumor cells and are supported by successful precedents in targeted cancer therapy.

To better understand the mechanisms driving its cytotoxicity, additional studies involving cell cycle assays, cell migration, invasion, angiogenesis, and gene expression profiling are warranted. These analyses will help determine the selectivity and mode of action of BamazScplp1 at the molecular level.

Elucidating the structural features of the peptide is also essential, particularly the disulfide bond arrangement, which is critical for stability and function. Once a more complete sequence is obtained, computational modeling can aid in predicting disulfide connectivity based on cysteine spacing and evolutionary conservation. These predictions may then be experimentally validated via mass spectrometry of reduced and non-reduced samples, followed by differential alkylation. High-resolution methods such as NMR or X-ray crystallography could further clarify the peptide’s three-dimensional structure and structure–activity relationships.

Altogether, this work underscores the biomedical relevance of exploring neglected venom sources and lays the foundation for the preclinical development of scorpion-derived peptides as therapeutic agents.

## Data Availability

The original contributions presented in the study are included in the article/Supplementary Material, further inquiries can be directed to the corresponding author.

## Supplementary material

SUPPLEMENTARY FIGURE S1 Validation of cell death induction using Paclitaxel in breast cancer cell lines. MCF10A, SKBR3, MCF7, and MDA-MB-231 cell lines (seeded at 1 × 10^5^ cells/well) were treated and incubated with Paclitaxel (853.9 g/mol) at concentrations of 0.01, 0.1, 1, 10, 100 and 1,000 μM for 24 h. Then, cell viability was assessed using the MTT assay. Data represent the mean ± SD of triplicate measurements from 2 independent experiments. NC - negative control (only culture medium). PC - positive control (30% DMSO). Statistical significance: group vs. NC, **p* < 0.05.

SUPPLEMENTARY FIGURE S2 Stepwise fractionation of *Brotheas amazonicus* venom using ultrafiltration. Crude venom (40 mg) was dissolved in 10 mL ultrapure water (18.2 MΩ cm, Synergy^®^ UV, Millipore, United States) and centrifuged (8,000 *xg*, 10 min, 4 C). The supernatant was collected, while the pellet was resuspended in 10 mL ultrapure water and centrifuged under identical conditions. This process was repeated three times, and all four supernatants were pooled. The combined supernatant was ultrafiltered using an Amicon^®^ Ultra-15 Centrifugal Filter Ultracel^®^ 10K, retaining components >10 kDa. The <10 kDa filtrate was subsequently processed with an Amicon^®^ Ultra-15 Centrifugal Filter Ultracel^®^ 3K, yielding two additional fractions: 3–10 kDa (retained) and <3 kDa (filtrate).

SUPPLEMENTARY FIGURE S3 Antitumor activity of FPLC peaks from Bamaz>10 fraction. Peaks P9, P27, P28, and P32 were tested for antitumor effects in breast cancer cell lines. MCF10A, SKBR3, MCF7, and MDA-MB-231 cell lines (seeded at 1 × 10^5^ cells/well) were treated and incubated with the indicated doses (see graphs) for 24 h. Then, cell viability was assessed using the MTT assay. Data represent the mean ± SD of triplicate measurements from 4 independent experiments. NC - negative control (only culture medium). PC - positive control (30% DMSO). Statistical significance: group vs. NC, **p* < 0.05.

SUPPLEMENTARY FIGURE S4 Antitumor activity of FPLC peaks from Bamaz>10 fraction. Peaks P36, P57, P77, and P85 were tested for antitumor effects in breast cancer cell lines. MCF10A, SKBR3, MCF7, and MDA-MB-231 cell lines (seeded at 1 × 10^5^ cells/well) were treated and incubated with the indicated doses (see graphs) for 24 h. Then, cell viability was assessed using the MTT assay. Data represent the mean ± SD of triplicate measurements from 3 independent experiments. NC - negative control (only culture medium). PC - positive control (30% DMSO). Statistical significance: group vs. NC, **p* < 0.05.

SUPPLEMENTARY FIGURE S5 MALDI-TOF/TOF spectrum of the internal peptide EQYFHK from BamazScplp1 obtained by *de novo* sequencing. Representative MS/MS spectrum showing the fragmentation pattern of the internal peptide EQYFHK derived from in-gel tryptic digestion of the predominant protein band in peak 59. Ions were detected in positive reflector mode following external calibration with Bruker Peptide Calibration Standard. The b- and y-ion series enabled confident assignment of the amino acid sequence. This spectrum supports the identification of BamazScplp1 and is consistent with the partial sequence derived by *de novo* analysis.

SUPPLEMENTARY FIGURE S6 Cytometry analysis strategy for cell death. Samples in the negative control condition and treated with BamazScplp1 were evaluated for morphology (FSC-A and SSC-A), single cells (FSC-A and FSC-H), and cell death (FVS and AnnexinV).
