# Supplementary figures and images for: A novel scorpine-like peptide from the amazonian scorpion Brotheas amazonicus with cytolytic activity
Source: Front Pharmacol. 2025 Sep 3;16:1652614. doi: 10.3389/fphar.2025.1652614 (PMC12441280; doi:10.3389/fphar.2025.1652614)

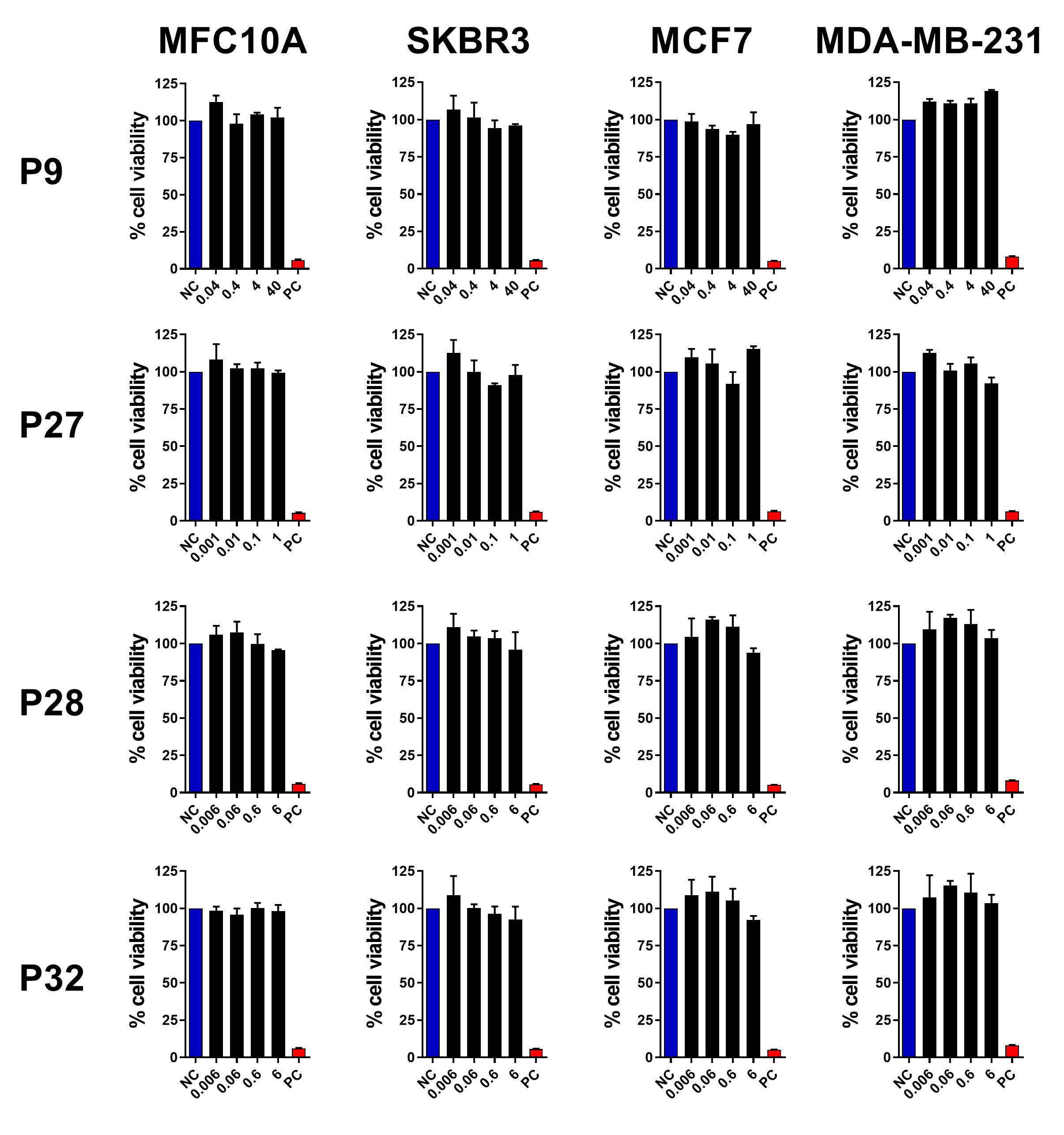

Supplement: Supplementary file 1 [file Image3.tif]

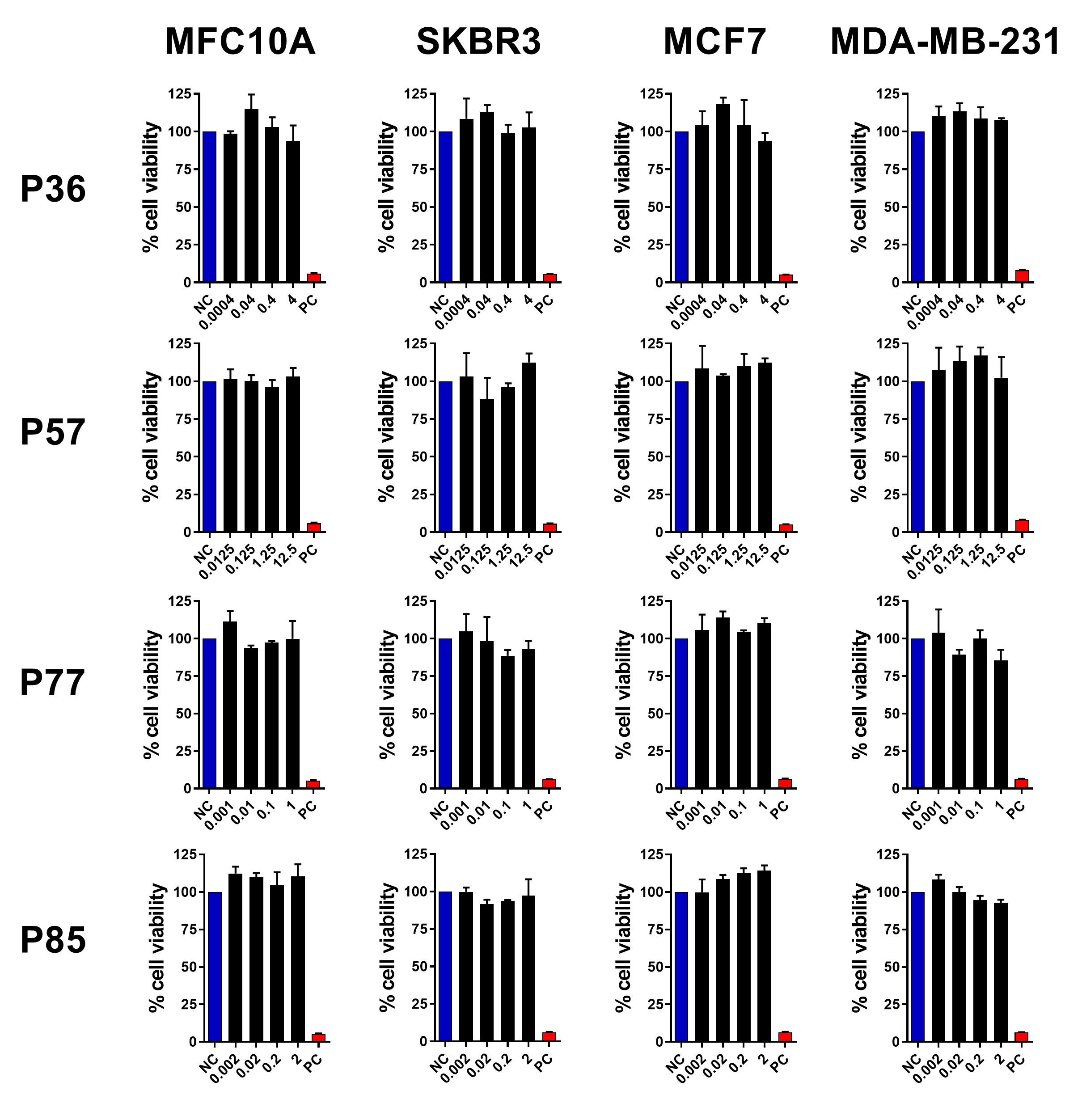

Supplement: Supplementary file 2 [file Image4.tif]

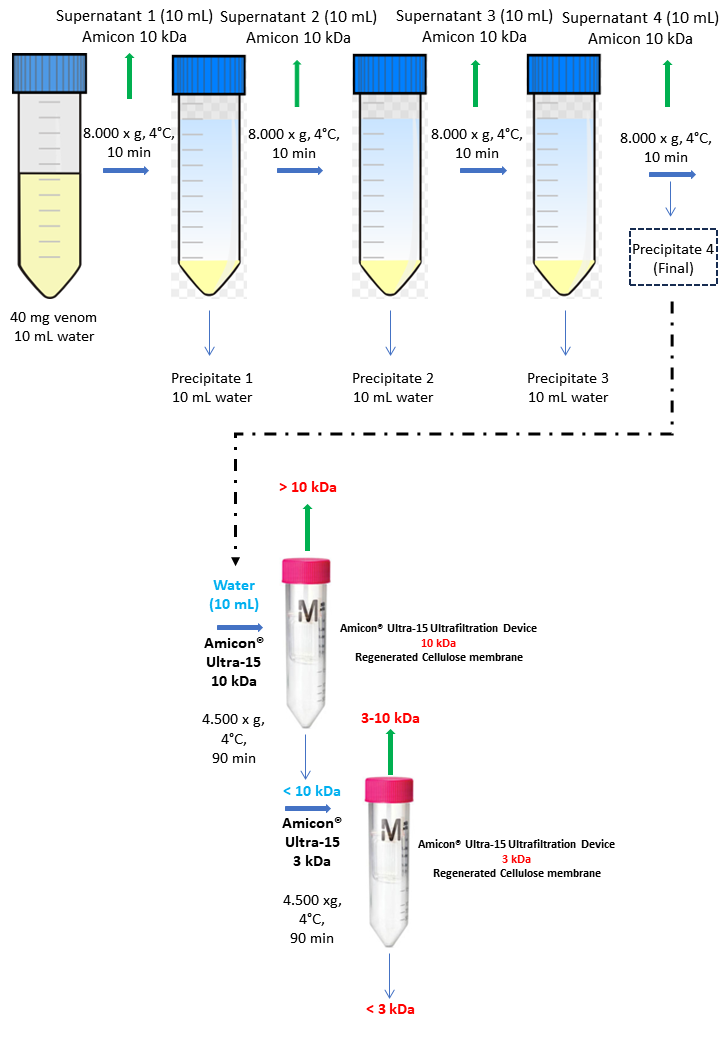

Supplement: Supplementary file 3 [file Image2.tif]

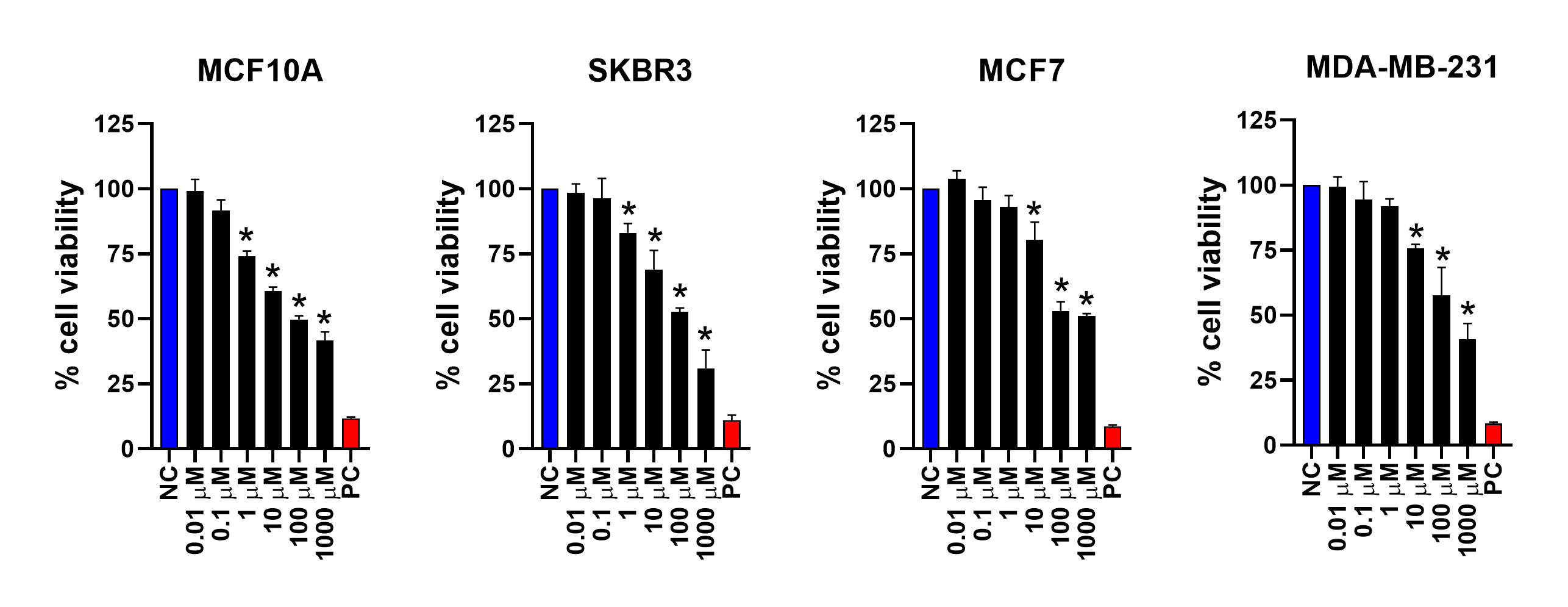

Supplement: Supplementary file 4 [file Image1.tif]

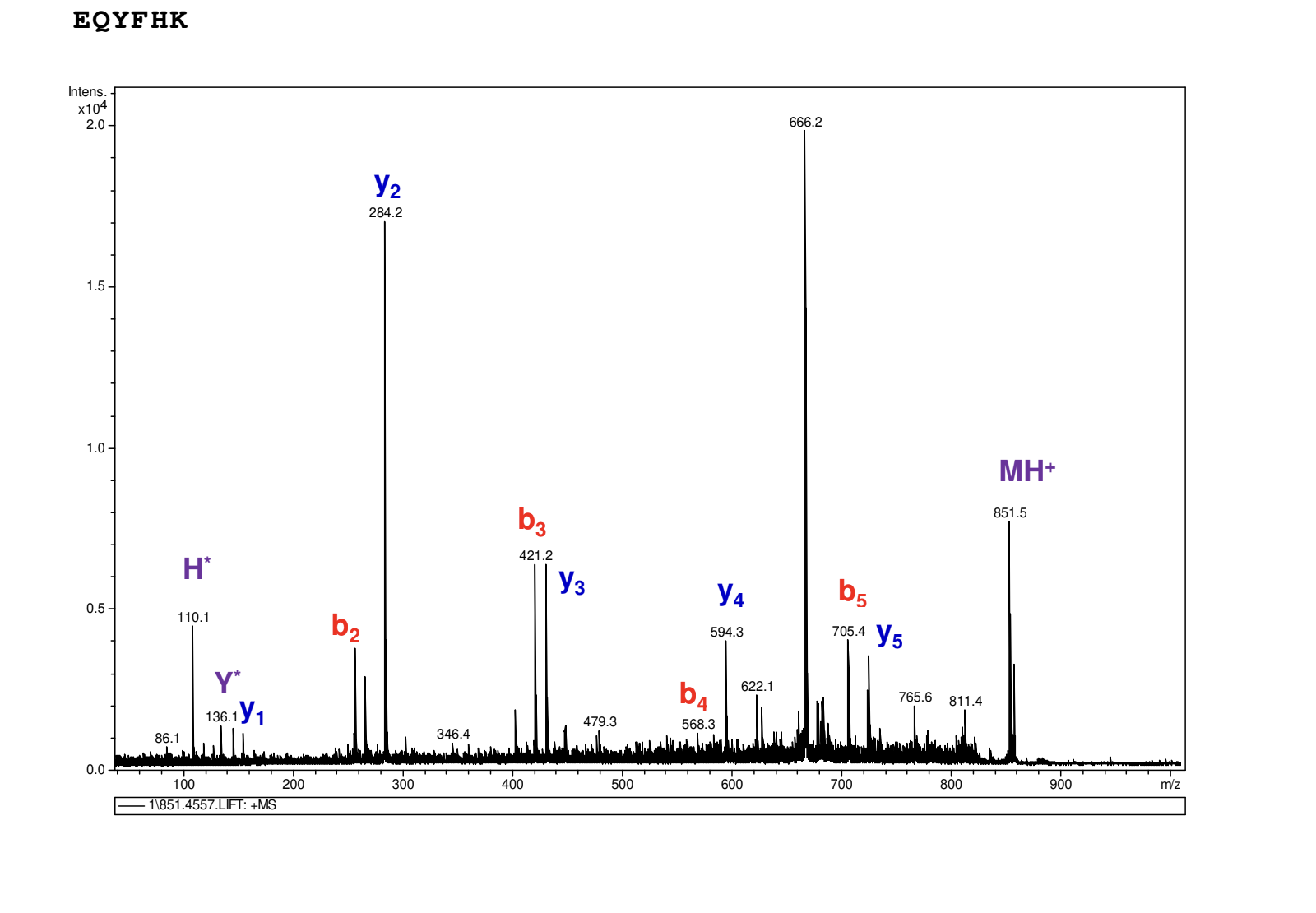

Supplement: Supplementary file 5 [file Image5.png]

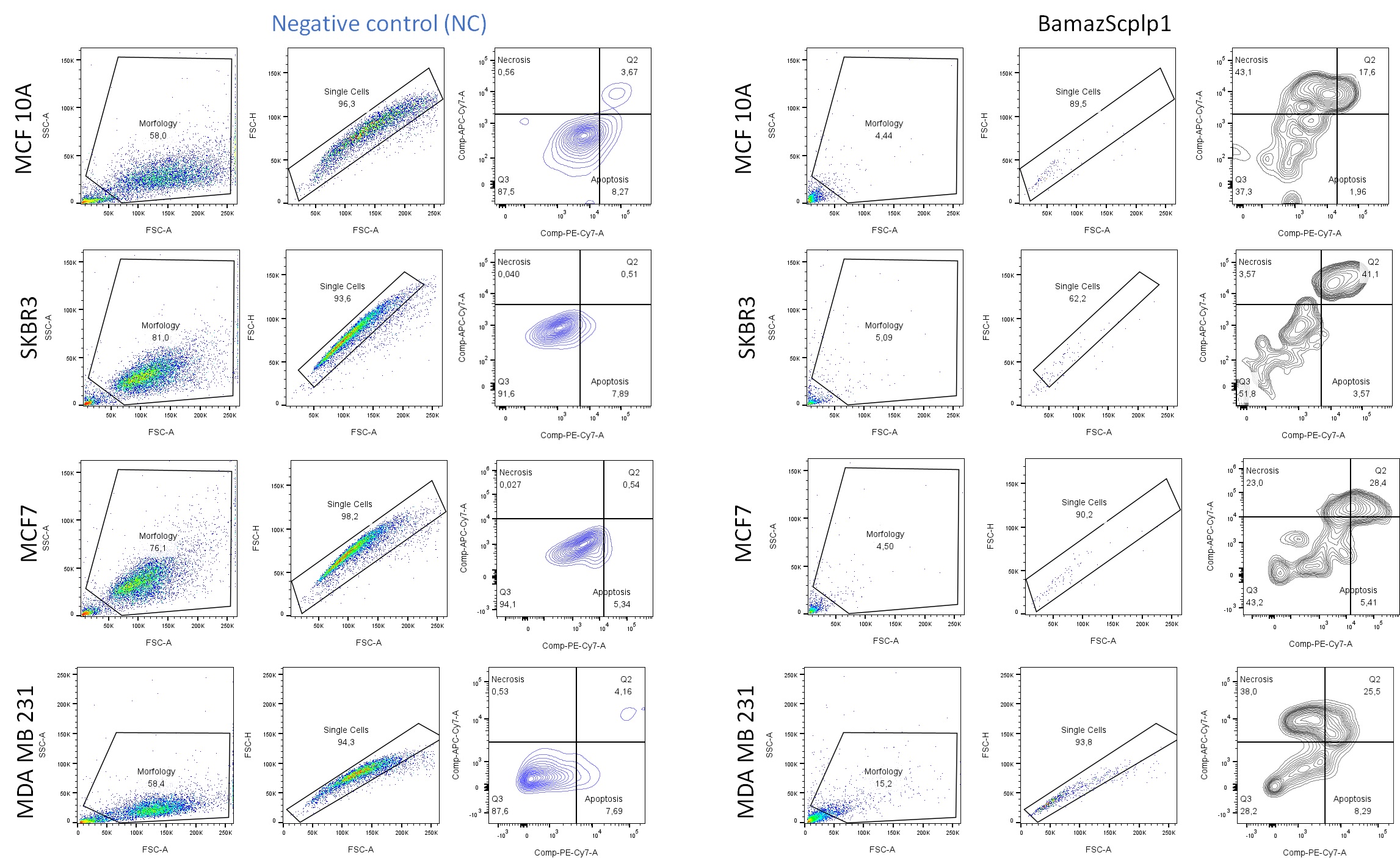

Supplement: Supplementary file 6 [file Image6.jpeg]
